# Impact of hormonal modulation at proestrus on ovarian responses and uterine gene expression of suckled anestrous beef cows

**DOI:** 10.1186/s40104-017-0211-3

**Published:** 2017-11-01

**Authors:** Manoel Francisco de Sá Filho, Angela Maria Gonella-Diaza, Mariana Sponchiado, Marcio Ferreira Mendanha, Guilherme Pugliesi, Roney dos Santos Ramos, Sónia Cristina da Silva Andrade, Gustavo Gasparin, Luiz Lehmann Coutinho, Marcelo Demarchi Goissis, Fernando Silveira Mesquita, Pietro Sampaio Baruselli, Mario Binelli

**Affiliations:** 1 Departamento de Reprodução Animal, FMVZ-USP, São Paulo, SP Brazil; 2 Departamento de Genética e Biologia Evolutiva, IB-USP-, São Paulo, SP Brazil; 3 Laboratório de Biotecnologia Animal, ESALQ-USP, Av Pádua Dias, Piracicaba, SP 11 Brazil; 4 Universidade Federal do Pampa, Uruguaiana, RS Brazil; 5 Universidade de São Paulo, Faculdade de Medicina Veterinária e Zootecnia, Departamento de Reprodução Animal, Avenida Duque de Caxias Norte, 225, Pirassununga, SP Zip Code 13635900 Brazil

**Keywords:** Cattle, eCG, Endometrium, Estradiol, Transcriptome

## Abstract

**Background:**

This study evaluated the impact of hormonal modulation at the onset of proestrus on ovarian response and uterine gene expression of beef cows.

**Methods:**

A total of 172 anestrous beef cows were assigned to one of four groups according to the treatment with estradiol cypionate (ECP) and/or equine chorionic gonadotropin (eCG) [CON (*n* = 43), ECP (n = 43), eCG (*n* = 44) and ECP + eCG (*n* = 42)].

**Results:**

ECP-treated cows (ECP and ECP + eCG groups) presented greater occurrence of estrus (44.6% vs. 65.4%; *P* = 0.01) and pregnancy per AI [47.1% vs. 33.3%; *P* = 0.07], but similar progesterone (P4) concentration at subsequent diestrus than cows not treated with ECP (CON and eCG groups). Nonetheless, eCG-treated cows (eCG and ECP + eCG groups) presented larger follicle at timed AI (12.6 ± 0.3 vs. 13.5 ± 0.3 mm; *P* = 0.03), greater ovulation rate (96.5% vs. 82.6%; *P* = 0.008) and greater P4 concentration at d 6 (3.9 ± 0.2 vs. 4.8 ± 0.2 ng/mL; *P* = 0.001) than cows not treated with eCG (CON and ECP groups). Next, cows with a new corpus luteum 6 d after TAI were submitted to uterine biopsy procedure. Uterine fragments [CON (*n* = 6), ECP (n = 6)] were analyzed by RNA-Seq and a total of 135 transcripts were differentially expressed between groups (73 genes up-regulated by ECP treatment). Subsequently, uterine samples were analyzed by qPCR (genes associated with cell proliferation). ECP treatment induced greater abundance of *PTCH2* (*P* = 0.07) and *COL4A1* (*P* = 0.02), whereas suppressed *EGFR* (*P* = 0.09) expression. Conversely, eCG treatment increased abundance of *HB-EGF* (*P* = 0.06), *ESR2* (*P* = 0.09), and *ITGB3* (*P* = 0.05), whereas it reduced transcription of *ESR1* (*P* = 0.05). Collectively, supplementation with ECP or eCG at the onset of proestrous of anestrous beef cows influenced ovarian responses, global and specific endometrial gene expression.

**Conclusion:**

Proestrus estradiol regulate the endometrial transcriptome, particularly stimulating proliferative activity in the endometrium.

**Electronic supplementary material:**

The online version of this article (10.1186/s40104-017-0211-3) contains supplementary material, which is available to authorized users.

## Background

Synchronization of estrus and ovulation programs for timed artificial insemination (TAI) has been constantly incorporated on modern reproductive management of beef farms [1, 2]. These programs can induce the first postpartum ovulation and, consequently, hasten the establishment of pregnancy of suckled beef cows [1, 3–5]. However, a significant proportion of ovulated and inseminated cows are detected not-pregnant 30 d after insemination despite the satisfactory ovulation rate (~85%) following protocols for synchronization of ovulation [4, 6, 7]. The uterine environment plays a relevant role among factors that are likely to contribute to the observed failures [8–10].

Early classic studies demonstrated the significant impact of a coordinated and sequential exposure to ovarian steroids on uterine function [11–13]. Gene expression of bovine endometrium changes according to the phase of the estrous cycle and is closely controlled by circulating concentrations of estradiol (E2), progesterone (P4) and the expression ratio of their specific receptors [8, 14–17]. In this regard, proestrus E2 concentration is fundamental in modulation of the uterus for the subsequent luteal phase [8, 14, 18, 19]. This E2 priming may be important for induction of endometrial P4 receptors [20, 21] to avoid premature luteolysis and short cycles in beef cattle [22]. In cyclic dairy heifers, elevated E2 concentrations during proestrus, induce changes in uterine gene expression of E2 and P4 receptors (*ESR1* and *PGR*, respectively), oxytocin receptors, and expression of cyclooxygenase-2, and beta subunit inhibin serpin-14 throughout the subsequent estrus cycle [23]. Also, cyclic beef heifers that are exposed to a longer proestrus period exhibit alterations in the pattern of steroids receptors expression in the uterus and other proteins associated with uterine receptivity to pregnancy [19]. Therefore, it is reasonable to hypothesize that the modulation of E2 concentration during the synchronized proestrus by means of exogenous E2 supplementation could also alter the uterine gene expression of suckled anestrous beef cows.

Two pharmacological strategies to manipulate the proestrus phase have been extensively evaluated in cattle breeding programs; exogenous E2 supplementation or equine chorionic gonadotropin (eCG) administration. Firstly, exogenous E2 supplementation using E2 esters enhances the proportion of cows that display estrus [24–26], increases endometrial thickness in lactating dairy cows [27] and improves the pregnancy success of suckled beef cows [6, 25, 26]. Furthermore, Jinks et al. [28] demonstrated that, recipients beef cows with lower E2 concentration at periovulatory phase, receiving in vivo-produced embryo, presented a dramatic reduction on pregnancy establishment (45% vs. 65% of pregnancy rate). Secondly, administration of eCG at onset of the proestrus is an efficient alternative to increase final follicular growth, ovulation rate and plasma P4 concentration on subsequent diestrus [5, 26, 29, 30]. Such changes may be responsible for the increase in pregnancy rates of anestrous beef cows stimulated with eCG [5, 26, 29, 30]. Altogether, both pharmacological strategies to manipulate the proestrus are capable of altering the periovulatory steroidal endocrine profiles, potentially modulating the expression of genes associated with uterine receptivity and ultimately positively influencing pregnancy establishment of suckled anestrous beef cows.

Therefore, based on the importance of the proestrus hormonal milieu on fertility, we hypothesized that supplementation with estradiol cypionate (ECP) and/or eCG at the onset of proestrus alters the ovarian response and the uterine transcriptome of suckled anestrous beef cows. To assess the above mentioned hypothesis, we chose the following approaches. First, taking a comprehensive approach, RNA extracts from endometrial fragments were submitted to Next Generation RNA sequencing followed by functional enrichment analysis to potentially identify and characterize other ECP-regulated biological and molecular processes and pathways. Secondly, following a candidate gene approach, we tested the effect of ECP and/or eCG supplementation on the expression of selected molecules with relevant biological functions in the context of uterine biology, specifically associated with cell proliferation.

## Methods

### Animals

Animal procedures were approved by the Ethics and Animal Handling Committee of the Faculdade de Medicina Veterinária e Zootecnia, Universidade de São Paulo (CEUA-FMVZ/USP, No. 2287/2011). This experiment was conducted during the 2012/2013 spring-summer breeding seasons. A total of 172 suckled anestrous Nelore (*Bos indicus*) beef cows at 30–60 d postpartum from a commercial farm in the state of Parana, Brazil, were enrolled in this study. Cows were maintained on *Brachiaria brizantha* pasture with water and mineral supplementation ad libitum. Immediately prior to the initiation of the TAI protocol, information about body condition score from each cow were recorded (BCS; range, 1 = emaciated to 5 = obese; with 0.5 scale) [31].

### Reproductive management and experimental design

**Fig. 1:**
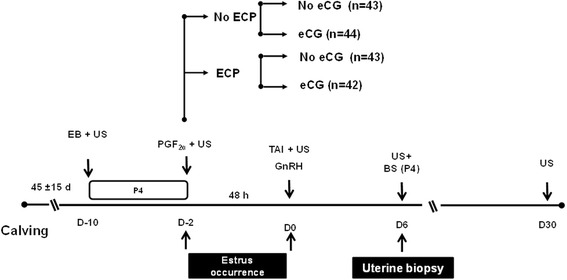
Schematic diagram of the synchronization of ovulation protocol in suckled anestrous beef cows. EB = 2 mg of estradiol benzoate; P4 = progesterone; P4 insert = previously used intravaginal P4 insert containing 1.0 g of P4; GnRH = 100 mg of gonadorelin; ECP = 1 mg of estradiol cypionate; eCG = 400 IU of equine chorionic gonadotropin; PGF2α = 0.25 mg of cloprostenol; US = ultrasound examination; BS = blood sample. Cows from ECP group received ECP and cows from eCG group received eCG, while cows from CON did not receive any further treatment and cows from ECP + ECG received both treatments

After calving, cows were allocated into breeding groups according to calving date. At 30 to 60 d post-partum, females were synchronized using an E2-plus-P4-based TAI protocol. Briefly, suckled cows received an intravaginal P4-releasing insert previously used for 8 d (1 g of P4; DIB®, MSD Animal Health, São Paulo, Brazil) on D −10 along with an intramuscular (IM) administration of 2 mg estradiol benzoate (EB; Gonadiol®, MSD Animal Health, São Paulo, Brazil; Fig. 1). The P4 insert were removed eight day later (D −2). All cows received an intramuscular administration of 500 mg of cloprostenol (Ciosin®, MSD Animal Health, São Paulo, Brazil) at the moment of the P4 insert removal. At this moment, cows were blocked by BCS, parity (multiparous vs. primiparous) and the diameter of the largest follicle and then randomly assigned into one of four experimental groups [Control (CON): *n* = 43, Estradiol cypionate (ECP): n = 43, eCG: *n* = 44, and ECP + eCG: *n* = 42], in a 2 × 2 factorial arrangement. Cows from ECP group received an IM injection of 1 mg of ECP (E.C.P.; Zoetis, São Paulo, Brazil), cows from eCG group received an IM injection of 400 IU of eCG (Folligon®, MSD Animal Health), while cows from ECP + eCG group received both treatments and cows from CON group did not receive any treatment. In all groups, ovulation was induced by 10 μg of buserelin acetate (GnRH, Sincroforte, Ourofino Saúde Animal, Cravinhos, São Paulo, Brazil) IM administration 48 h after the P4 insert removal (D 0). Cows were artificially inseminated immediately after GnRH treatment. Inseminations were performed by a single technician using frozen-thawed semen from single Angus sire with proven fertility. The sire used had been previously used in TAI programs and had satisfactory (~50%) pregnancy results.

Estrus was determined based on the tail-head mark. At the time of the removal of the P4 insert, the tail-head was marked with chalk (Raidl-Maxi, RAIDEX GmbH, Dettingen/Erms, Germany). Estrus was deemed to have occurred in cattle without a tail-head mark at TAI.

Cows presenting a corpus luteum (CL) on D 6 (6 d after GnRH treatment) had the body of the uterus biopsied as previously described [32]. Fragments obtained from uterine biopsies were individually allocated in cryotubes and immediately immersed into liquid nitrogen. Day 6 was strategically selected as the moment in which an early embryo is expected to have recently accessed the uterine environments. Pregnancy was diagnosed by transrectal ultrasonography through the detection of a viable embryo (presence of heartbeat) on d 42 post-AI.

### Blood sampling and hormone measurements

Blood sampling for determination of P4 concentrations was performed on D 6, concurrently with the uterine biopsy. Blood samples were collected by coccygeal venipuncture using evacuated tubes containing EDTA (BD, São Paulo, SP, Brazil) and immediately stored in ice. Plasma was separated by centrifugation at room temperature, 1,500 × g for 15 min, and stored at −20 °C. Progesterone concentrations were measured in all samples using a solid-phase radioimmunoassay (Coat-a-count, Siemens, Los Angeles, USA), as validated previously [33]. The P4 assay sensitivity was 0.08 ng/mL and the intra-assay coefficient of variation was 8.7%.

### Ultrasound examinations

Transrectal ultrasound examinations were carried out on D −10, D −2, D 0 and D 6 to assess cyclic status, growth of the dominant follicle (DF), ovulation, and the presence of CL. Ultrasonography was performed with the aid of a B-mode (gray-scale) ultrasound instrument (8100, Chison Medical Imaging, Co, China), equipped with a multi-frequency linear-array transducer. The anestrous status was defined as the absence of CL in two consecutive ultrasound examinations performed on D − 10 and D −2. Ovulation was defined as the presence of a recently formed CL on D 6 on the same ovary that the DF was observed on D −2 and D 0. The diameter of the DF at the time of P4 insert removal and at TAI, in addition to the diameter of new CL formed, was calculated as the average between measurements of two perpendicular axes of each structure.

### Tissue processing, RNA isolation and cDNA synthesis

Approximately 30 mg of endometrial tissue was macerated in liquid nitrogen using a stainless steel mortar and pestle and immediately mixed with buffer RLT from the PureLink®, RNA Mini kit (Thermo Fisher Scientific, São Paulo, SP, Brazil), as per manufacturer’s instructions. To maximize lysis, tissue suspension was passed at least ten times through a 21-ga needle, and centrifuged at 13,000 × g for 3 min for removal of debris, prior to supernatant loading and processing on RNeasy columns. Columns were eluted with 40 μL of RNase free water. Concentration of total RNA on extracts was measured by a spectrophotometer (Nanovue™ Plus, Spectrophotometer, GE Healthcare, UK, by the absorbance at 260 nm). Subsequently, samples were treated with 80 μL of DNAse I solution (Life Technologies, São Paulo, SP, Brazil) for 15 min at room temperature during RNA extraction protocol, according to manufacturer’s instructions. RNA samples were stored at −80 °C until cDNA synthesis. The cDNA was synthesized by reverse-transcription using High Capacity cDNA Reverse Transcription Kit (Life Technologies) according to manufacturer’s instructions. Briefly, 10 μL of master mix containing RT buffer, dNTP mix, random primers, RNase inhibitor and reverse transcriptase were mixed to 1 μg of total RNA and final volume of the reaction was adjusted to 20 μL. Immediately, reactions were incubated at 25 °C for 10 min, followed by incubation at 37 °C for 2 h, and reverse-transcriptase inactivation at 85 °C for 5 min. Samples were stored at −20 °C.

### RNAseq

Prior to the RNA-seq analyses, 12 samples (*n* = 6/group; ECP and CON) were selected according to previously established criteria by ovarian, occurrence of estrus, pregnancy and endocrine responses. Cows having similar DF diameter at the time of P4 insert removal [ECP (12.1 ± 0.7 mm) and CON (12.1 ± 0.6 mm)] and similar circulating P4 concentration at the time of uterine biopsy [ECP (3.8 ± 0.2 ng/mL) and CON (3.6 ± 0.2 ng/mL)] were considered suitable to further analysis. Additionally, cows were also selected based on pregnancy status 30 d after TAI in order to have both pregnant and non-pregnant cows represented in both experimental groups. Finally, only cows displaying estrus were selected in ECP treated group, whereas only cows that did not display estrus were chosen in the control group. The latter criterion was applied aiming to increase the distinction between two different E2 pre-ovulatory endocrine environments, as cows that display estrus present greater E2 concentration than those not displaying estrus [42].

Integrity of total RNA extracts was assessed using the Agilent RNA 6000 Nano chip (Bioanalyzer, Agilent Technologies). RNA Integrity Number (RIN) of extracts submitted to RNA sequencing analysis ranged from 8.3 to 8.7. Next, 4 μg of RNA were used with the TruSeq RNA Sample Preparation kit (Illumina, San Diego, CA) to prepare the libraries for RNA-Seq. The insert sizes were estimated through the Agilent DNA 1000 chip (Agilent Technologies) and the libraries concentration were measured through Quantitative Real-Time PCR (qPCR) with a KAPA Library Quantification kit (KAPA Biosystems). Samples were diluted, pooled in equimolar amounts and then sequenced at the Centro Genômico Funcional Aplicado a Agropecuária e Agroenergia at the University of São Paulo using a HiScanSQ sequencer (Illumina, San Diego, CA).

### Bioinformatics analyses

Raw sequences were trimmed for adaptors and low quality using SeqyClean v1.3.12. (https://github.com/ibest/seqyclean) using 26 Phred quality parameter for maximum average error and a fasta file with contaminant sequences from the Univec database (https://www.ncbi.nlm.nih.gov/tools/vecscreen/univec/). Only high quality paired-end sequences were kept for further analyses The reads were mapped with Bowtie2 v2.1.0 [34] on the masked bovine genome assembly (*Bos taurus* UMD 3.1, NCBI). The mapping file was sorted using SAMTools v 0.1.18 [35] and read counts were obtained using the script from HTSeq-count v0.5.4p2 (http://htseq.readthedocs.io/en/release_0.9.1/). The differential expression analysis was performed with package DESeq2 [36] from R [37]. Using the function estimateSizeFactors, the normalized counts were obtained (baseMean values, which are the number of reads divided by the size factor or normalization constant). The standard deviation along the baseMean values was also calculated for each gene. In order to avoid artifacts caused by low expression profiles and high expression variance, only transcripts that had an average of baseMean >5 and the mean greater than the standard variation were analyzed. The threshold for evaluating significance was obtained by applying an alpha ≤0.10, considering the FDR-Benjamini-Hochberg *P*-value [38]. Integrated analysis of different functional databases was done using the functional annotation tool of the Database for Annotation, Visualization, and Integrated Discovery using as background the genes (DAVID) [39] using as background the set of genes that passed through the differential expression analysis filter.

### qPCR

The samples employed in qPCR analysis were selected mirroring the general results obtained in regard to ovarian and endocrine responses. Cows receiving ECP should present greater occurrence of estrus, while cows from eCG treatment group should present greater circulation of P4 concentration at the moment of the uterine biopsy. Step-One Plus thermocycler (Life Technologies, Carlsbad, CA) and SYBR Green chemistry were used for quantitative PCR analysis. Primers were designed based on the mRNA sequence of target genes obtained from the RefSeq database, on Genbank (http://www.ncbi.nlm.nih.gov/genbank/). Sequences were masked to remove repetitive sequences with RepeatMasker (http://www.repeatmasker.org/) [40] and then, the masked sequences were used for primer design using the PrimerQuest software (IDT1, http://www.idtdna.com/primerquest/Home/Index). The characteristics of the primers were checked in Oligo Analyzer 3.1 software (IDT1, http://www.idtdna.com/analyzer/Applications/OligoAnalyzer/), while the specificity was compared by BLAST (NCBI, http://blast.ncbi.nlm.nih.gov). The qPCR products obtained from reactions performed with primers not previously validated were submitted to agarose gel electrophoresis and SANGER-DNA sequencing, and identities of target genes were confirmed. Details of primers are provided on Table 1. In order to select reference genes, the GeNorm Microsoft Excel applet was used, as this applet provides a measure of gene expression stability (M) [41]. The Glyceraldehyde-3-Phosphate Dehydrogenase (*GAPDH*), Actin, Beta (*ACTB*) and Ribosomal Protein S18 (*RPS18*) were the most stable genes and were, therefore, selected as reference genes. Determination of qPCR efficiency and Cq (quantification cycle) values per sample were performed with LinRegPCR software (V2014.2; http://www.hartfaalcentrum.nl/index.php?main=files&fileName=LinRegPCR.zip&description=LinRegPCR:%20qPCR%20data%20analysis&sub=LinRegPCR). Quantification was obtained after normalization of the target genes expression values (Cq values) by the geometric mean of the endogenous control expression values. The following genes, associated with regulation of cell proliferation in the uterus, were selected: ovarian steroid receptors [Estrogen Receptor alpha (*ESR1*), Estrogen Receptor beta (*ESR2*), P4 receptor (*PGR*)], growth factors that regulate cellular proliferation [epidermal growth factor receptor (*EGFR*), heparin-binding EGF-like growth factor (*HB-EGF*) and patched homolog 2 (*PTCH2*)], and extracellular matrix [collagen, type IV, alpha 1 (*COL4A1*) and integrin, beta 3 (*ITGB3*)].

**Table 1:** Gene name, accession number, forward and reverse primer sequences used for qPCR analysis

Gene Name	Gene ID	Sequence ID	Forward primer sequence (5′–3′)	Reverse sequence (5′–3′)	Primer efficiency, %	Amplicon length, bp
Progesterone receptor	*PGR*	NM_001205356.1	GCCGCAGGTCTACCAGCCCTA	GTTATGCTGTCCTTCCATTGCCCTT	96.9	199
Estrogen receptor 1	*ESR1*	NM_001001443.1	CAGGCACATGAGCAACAAAG	TCCAGCAGCAGGTCGTAGAG	99.1	82
Estrogen receptor 2	*ESR2*	NM_174051.3	TCACGTCAGGCACGCCAGTAAC	CACCAGGTTGCGCTCAGACCC	99.5	155
Patched 2^a^	*PTCH2*	XM_005197904.1	CATCCTGCTGCTGTGTACTT	ATCGCCAGGACCAGTACTAT	99.9	87
Epidermal growth factor receptor	*EGFR*	XM_002696890.3	ATGCTCTATGACCCTACCAC	TTCCGTTACAAACTTTGCCA	97.6	178
Heparin-binding EGF-like growth factor	*HB-EGF*	NM_001144090.1	CATCCACGGAGAATGCAAATAC	CAGCAGACAGACGGATGATAG	98.6	181
Collagen, type IV, alpha 1	*COL4A1*	NM_001166511.1	CACGGCTACTCTTTGCTCTAC	GAAGGGCATGGTACTGAACTT	96.48	102
Integrin, beta 3 (platelet glycoprotein IIIa, antigen CD61)	*ITGB3*	NM_001206490.1	GGGAGAGTGCTATGGTTAGA	CTTCACAAGACACCCAAGAG	92.09	142
Actin Beta	*ACTB*	NM_173979.3	GGATGAGGCTCAGAGCAAGAGA	TCGTCCCAGTTGGTGACGAT	93.7	77
Glyceraldehyde-3-Phosphate Dehydrogenase	*GAPDH*	NM_001034034.2	GCCATCAATGACCCCTTCAT	TGCCGTGGGTGGAATCA	99.99	69
Ribosomal Protein S18	*RPS18*	AY786141.1	TGGAGAGTATTGCGCCTTCTC	CACAAGTTCCACCACACTATTGG	97.9	79

^a^Transcript variants X1 to X7

### Statistical analyses from ovarian, endocrine and gene expression responses

The statistical analyses for ovarian responses were performed using the PROC GLIMMIX of SAS for Windows (SAS 9.3 Institute Inc., Cary, NC, USA, 2003). Continuous variables were presented as mean ± standard error of the mean (mean ± SEM) and percentage (%) for frequency of occurrence for binomial variables. The continuous response variables were subjected to response scaling test through the solution Guided Data Analysis of SAS. Variables that did not follow these assumptions were transformed accordingly. Binomial variables (i.e. occurrence of estrus and ovulation rate) were analyzed by logistic regression using the SAS GLIMMIX procedure with models fitted to binomial distributions. The explanatory variables considered for inclusion in the models were the treatment with ECP, eCG and interaction of ECP and eCG. The effect of cow within each replicate was included as a random effect.

The qPCR data were tested for normality of residuals and homogeneity of variances followed by ANOVA using the GLIMMIX procedure of SAS fitting log normal distribution. The explanatory variables considered for inclusion in the models were the treatment with ECP, eCG and interaction between ECP and eCG. Final results are presented in natural log (Ln) scale (because of the log normal distribution considered) as normalized values of a specific gene transcript by the mean level of the transcript from Control (No-ECP and No-eCG treated animals). Down-regulation of expression in a specific experimental group may be represented by negative values relative to control because of Ln scale. To avoid negative values, the mean used for data normalization was divided by the fifth negative exponent. All data were compared with the relative mean expression level of the control group.

Statistical difference was considered when *P* < 0.10. Graphs were plotted with Sigmaplot (version 11.0; Systat Software, Inc. San Jose, CA, USA).

## Results

### Ovarian, pregnancy and endocrine responses

Animals receiving different hormonal therapies at the proestrus presented different rates of occurrence of estrus between P4-releasing device removal and TAI, final follicular growth, ovulatory responses and subsequent CL function (Table 2). There were no interactions between ECP and eCG treatment on response variables, except for the CL diameter 6 d after the TAI (*P* = 0.06). Larger CLs were observed in cows treated with eCG, especially in cows not treated with ECP. The ECP treated cows presented a greater frequency of occurrence of estrus [ECP = 64.7% (55/85) vs. No-ECP = 44.8% (39/87); *P* = 0.008] and presented greater pregnancy per TAI [ECP = 47.1% (40/85) vs. No-ECP = 33.3% (29/87); *P =* 0.07]. Cows treated with eCG presented greater rate of final follicular growth [eCG = 1.2 ± 0.1 mm/d vs. No-eCG = 0.9 ± 0.1 mm/d; *P =* 0.01], resulting in a greater DF diameter at TAI [eCG = 13.5 ± 0.3 mm vs. No-eCG = 12.6 ± 0.3 mm; *P* = 0.03]. Also, a greater proportion of cows receiving eCG displayed estrus [eCG = 62.8% (54/86) vs. No-eCG = 46.5% (40/86); *P* = 0.03] and ovulated [eCG = 96.5% (83/85) vs. No-eCG = 82.6% (71/86); *P* = 0.008]. A greater P4 concentration at the moment of uterine biopsy (D 6) was observed in cows receiving eCG at the onset of the proestrus [eCG = 4.8 ± 0.2 ng/mL vs. No-eCG = 3.9 ± 0.2 ng/mL; *P =* 0.001]. However, there was no influence of eCG treatment on the pregnancy per TAI [eCG = 43.0% (37/86) vs. No-eCG = 37.2% (32/86); *P =* 0.42].

**Table 2:** Overall occurrence and effects of treatment with estradiol cypionate (ECP) and/or equine chorionic gonadotropin (eCG) at onset of the proestrus on follicular and luteal development in an estradiol/progesterone-based synchronization protocol on anestrous suckled beef cows

Itens	Treatments^1^				*P*value		
	No ECP		ECP				
	No eCG	eCG	No eCG	eCG	ECP	eCG	ECP × eCG
Number of cows	43	44	43	42	–	–	–
BCS at onset of the synchronization^2^	3.1 ± 0.1	3.0 ± 0.1	2.9 ± 0.1	3.0 ± 0.1	0.12	0.71	0.43
DF diameter at insert removal, mm^3^	11.0 ± 0.4	11.2 ± 0.4	10.8 ± 0.4	11.3 ± 0.4	0.90	0.38	0.77
DF diameter at TAI, mm^4^	12.6 ± 0.4	13.6 ± 0.4	12.7 ± 0.4	13.4 ± 0.4	0.90	0.03	0.68
Daily DF growth, mm/d^5^	0.9 ± 0.1	1.3 ± 0.1	0.9 ± 0.1	1.1 ± 0.1	0.52	0.01	0.25
Occurrence of estrus, %^6^	37.2	52.3	55.8	73.8	0.008	0.03	0.77
Ovulation rate, %^7^	81.4	95.5	83.7	97.6	0.54	0.008	0.71
CL diameter at d 6 after TAI, mm	17.8 ± 0.6^b^	20.1 ± 0.5^a^	18.6 ± 0.6^ab^	18.7 ± 0.6^ab^	0.54	0.04	0.06
Plasma P4 at d 6 after TAI, ng/mL	3.8 ± 0.3	5.1 ± 0.3	4.1 ± 0.3	4.6 ± 0.3	0.94	0.001	0.18
Pregnancy per TAI, %	30.2	36.4	44.2	50.0	0.07	0.42	0.95

^1^Suckled anestrous beef cows received an previously used intravaginal insert containing 1.0 g of progesterone (P4) and 2.0 mg of estradiol benzoate on the first day of the estrus/ovulation synchronization protocol (D −10). The P4 insert was removed eight days later (D −2), and cows from ECP group received an IM treatment of 1 mg of ECP, cows from eCG group received an IM injection of 400 IU of eCG, while cows from ECP + ECG received both treatments and cows from CON did not receive any treatment at this moment. All cows received GnRH IM and were timed artificially inseminated (TAI) 48 h after the P4 insert removal (D 0). Different letters within the same row indicate the presence of difference between groups (P <0.05) when an interaction between eCG and ECP was observed

^2^BCS = Body condition score collected at insertion of the P4 insert

^3^DF = Dominant follicle

^4^TAI = timed artificial insemination

^5^DF growth between the P4 insert removal and TAI divided by two

^6^Estrus determined based on the tail-head mark

^7^Number of cows with a new CL formed 6 d after the TAI divided by the number of animal synchronized

### RNA-seq

**Fig. 2:**
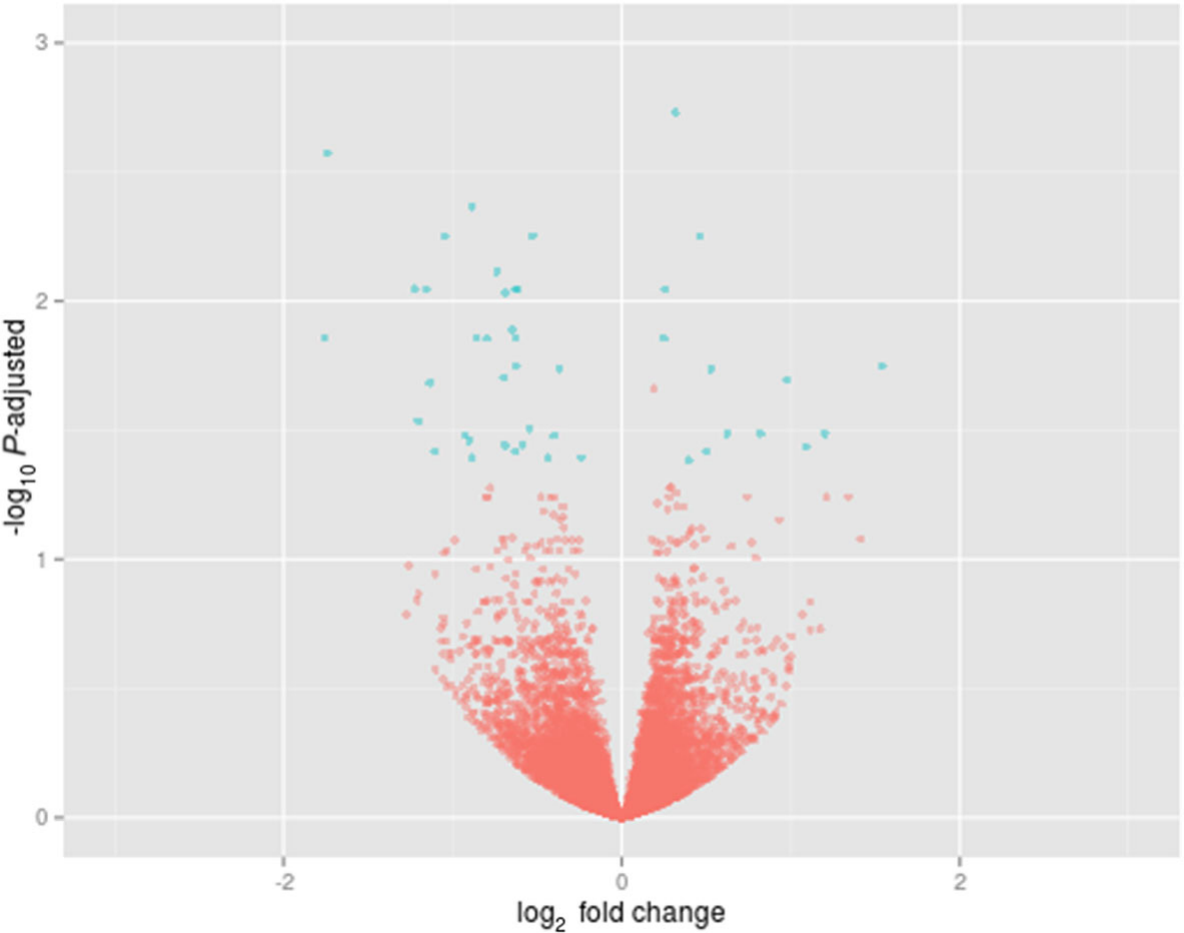
Volcano plot obtained from DESeq analysis. Volcano plot shows that the vertical lines axe is log_2_-fold change and the horizontal axis is the statistical significance (*P* value ≤ 0.10). Genes with *P* value ≤0.10 are marked with blue dots

**Fig. 3:**
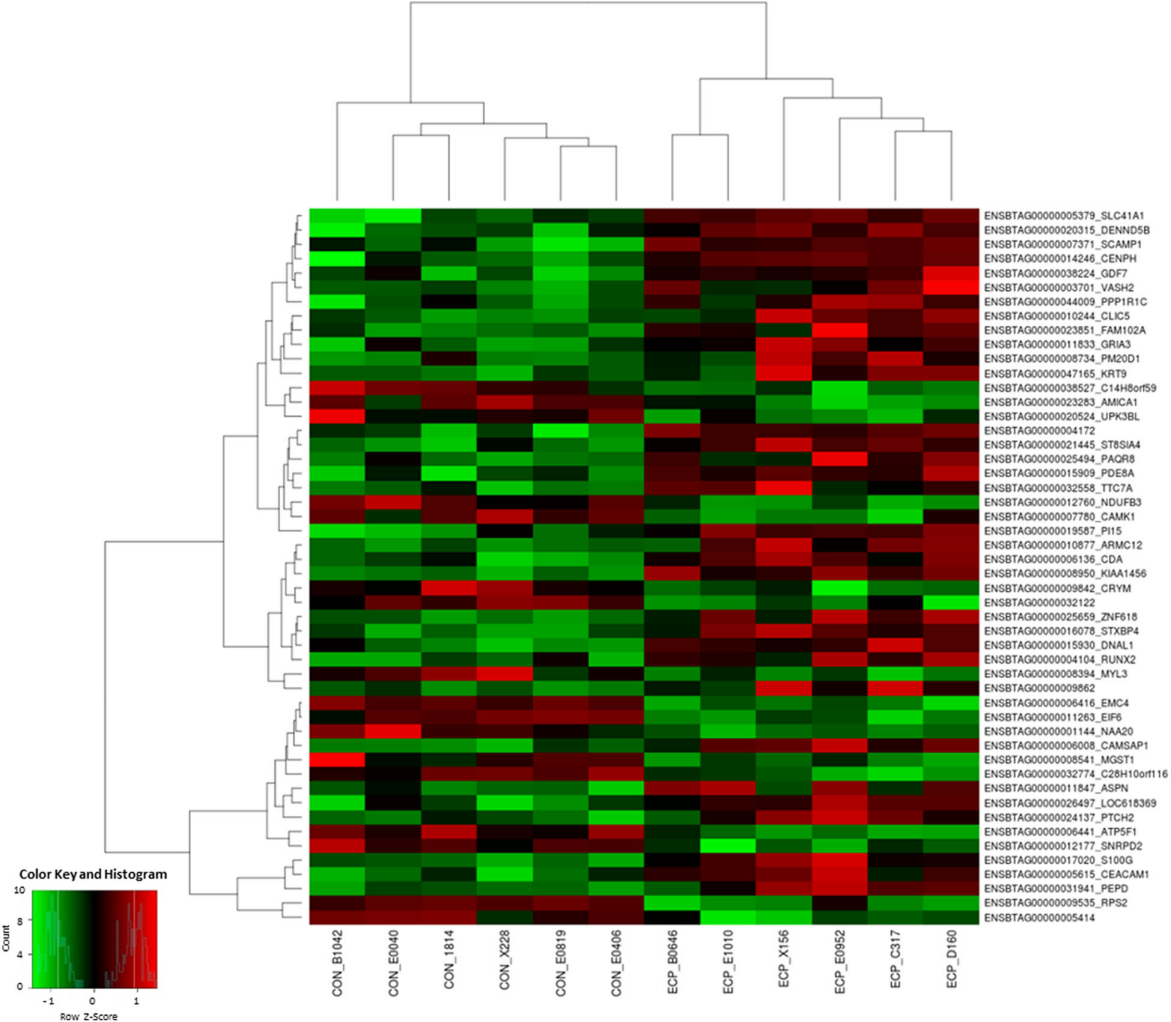
Heat map obtained from DESeq2 analysis. Each column represent one sample showing the intensity of expression profile per gene. The colors in the map display the relative standing of the reads count data; GREEN indicates a count value that is lower than the mean value of the row while red indicates higher than the mean. The shades of the color indicate distance from each data point to the mean value of the row

RNA sequencing produced a total of ~334 million reads with an average of 27.5 million reads for each group. Six biological replicates were analyzed for each phenotype (please see Statistical Analyses section above) with the reads ranging from 17 to 26 million per sample after filtering (Additional file 1: Table S1). Approximately ~65% of the total reads uniquely mapped to the UMD 3.1 reference genome (https://www.ncbi.nlm.nih.gov/genome?term=bos%20taurus). Only the uniquely mapped reads were considered in the analysis. From the remaining, approximately 20% of the reads were not uniquely mapped, and 15% unmapped reads. After applying the variance and minimal value of baseMean filtering, a total of 15,161 genes were included on the differential expression analysis. A total of 310 out of the 15,161 analyzed genes showed differential expression (adjusted *P*-value <0.1), of which 73 and 62 were upregulated in the endometrium of ECP and CON samples, respectively (see Volcano plot, Fig. 2 and Additional file 2: Table S2). Differentially expressed genes (DEG) with the greatest expression profiles were RPS2 [ribosomal protein S2], GABARAP [GABA (A) receptor-associated protein], up-regulated in the CON endometrium, and PEPD [peptidase D], SG100g [calcium binding protein G] and CEACAM1 [carcinoembryonic antigen-related cell adhesion molecule 1], up-regulated in the ECP group. Heatmap on Fig. 3 shows the 50 genes with the lowest p-adjusted values. It is possible to observe the similarity of gene expression patterns among individuals within each group, as indicated by the shades of green (for low expression) or red color (high expression).

Sequences of all reads were deposited in the Sequence Read Archive (SRA) of the NCBI (http://www.ncbi.nlm.nih.gov/sra/; Additional file 3: Table S3) and, an overview of these data has been deposited in NCBI’s Gene Expression Omnibus (GEO) and is accessible through GEO Series accession number GSE67807.

### Functional enrichment analysis of RNA-seq data - DAVID results

KEGG pathway and Gene ontology (GO) term analyses were performed with DAVID (Table 3). Functional enrichment analysis using DAVID revealed two KEGG pathways overrepresented by the ECP-upregulated transcripts: pathways in cancer (5 genes; *P <* 0.01) and small cell lung cancer (3 genes; *P* < 0.05). On the other hand, ECP downregulated transcripts indicated the enrichment of three pathways: Parkinson’s disease (3 genes; *P* = 0.06), oxidative phosphorylation (3 genes; *P* = 0.06) and Alzheimer’s disease (3 genes; *P* = 0.09). More specifically, ECP-upregulated transcripts associated with pathways in cancer were [gene symbol (fold change; adjusted *P* value on RNA-seq); respectively]: *LAMC3* (1.55; *P* = 0.10), *PTCH1* (1.51; *P* = 0.09), *PTCH2* (1.52; *P* = 0.03), *PIK3R3* (1.22; *P* = 0.10), and *PIAS1* (1.18; *P* = 0.09), whereas ECP downregulated transcripts associated with oxidative phosphorylation were *ATP5F1* (1.18; *P* = 0.01), *ATP5J* (1.24; *P* = 0.06), and *NDUFB3* (1.37; *P* = 0.01). Additionally, analysis of GO terms identified that ECP upregulated transcripts over represented epidermis development [*ADAM9* and ENSBTAG00000017455 (uncharacterized protein)]. On the other hand, ECP downregulated GO terms indicated the enrichment of five biological processes: generation of metabolic precursors and energy (*GPI*, *NDUFB3*, *ATP5F1*, *IDH3B*, *ATP5J*), Translation (*RPS2*, *EEF1D*, ENSBTAG00000013866, ENSBTAG00000011263), and mRNA processing, mRNA metabolic process and RNA splicing with 3 common genes (*GEMIN7*, *SNRPD2*, *STRAP*).

**Table 3:** KEGG Pathways and Gene ontologies (GO category) of mRNA transcripts differentially expressed in cows treated with estradiol cypionate (ECP).

Category	Term	Count	*P* value	Genes
Upregulated in ECP				
KEGG PATHWAY	Pathways in cancer	5	0.008	*PIK3R3, PTCH1, PTCH2, LAMC3, PIAS1*
KEGG PATHWAY	Small cell lung cancer	3	0.020	*PIK3R3, LAMC3, PIAS1*
GO TERM BP_FAT	Epidermis development	2	0.095	*ADAM9, ENSBTAG00000017455*
Downregulated in ECP				
KEGG PATHWAY	Parkinson’s disease	3	0.062	*NDUFB3, ATP5F1, ATP5J*
KEGG PATHWAY	Oxidative phosphorylation	3	0.064	*NDUFB3, ATP5F1, ATP5J*
KEGG PATHWAY	Alzheimer’s disease	3	0.090	*NDUFB3, ATP5F1, ATP5J*
GO TERM BP_FAT	Generation of precursor metabolites and energy	5	0.004	*GPI, NDUFB3, ATP5F1, IDH3B, ATP5J*
GO TERM BP_FAT	Translation	4	0.059	*RPS2*, *EEF1D*, *ENSBTAG00000013866, ENSBTAG00000011263*
GO TERM BP_FAT	mRNA processing	3	0.071	*GEMIN7, SNRPD2, STRAP*
GO TERM BP_FAT	mRNA metabolic process	3	0.087	*GEMIN7, SNRPD2, STRAP*
GO TERM BP_FAT	RNA splicing	3	0.041	*GEMIN7, SNRPD2, STRAP*

Enrichment analysis was performed with DAVID tools (https://david.ncifcrf.gov/tools.jsp)

### Cell proliferation-related gene expression

According to selection criteria described previously, uterine tissue used in the qPCR analysis derived from cows that presented different estrus responses [CON (10.1%), ECP (90.9%), eCG (66.7%) and ECP + ECG (83.3%)] and P4 concentration at uterine biopsy [CON (3.4 ± 0.2 ng/mL), ECP (3.7 ± 0.2 ng/mL), eCG (5.3 ± 0.4 ng/mL) and ECP + ECG (5.0 ± 0.6 ng/mL)].

**Fig. 4:**
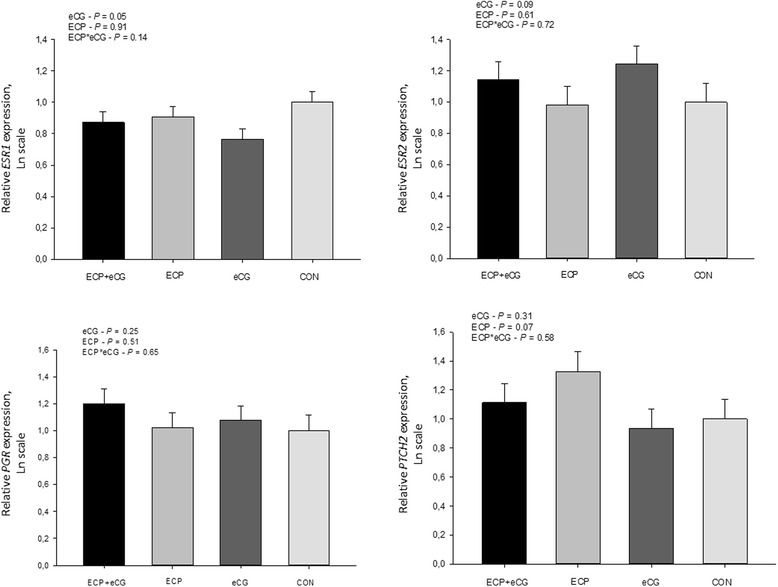
Comparison of gene expression between suckled anestrous beef cows receiving 1 mg of estradiol cipionate (ECP) and/or 400 IU of equine chorionic gonadotropin (eCG) at onset of the proestrus [CON (*n* = 11), ECP (n = 11), eCG (*n* = 12) and ECP + ECG (n = 11)]. The amounts of *ESR1*, *ESR2*, *PGR,* and *PTCH2* transcripts are expressed in relation to control (CON) untreated cows. Expression values were normalized by the geometric mean of *GAPDH*, *ACTB,* and *RPS18*. The *P* values refer to comparisons made for each gene between groups (effects of ECP, eCG and interaction between ECP and eCG)

**Fig. 5:**
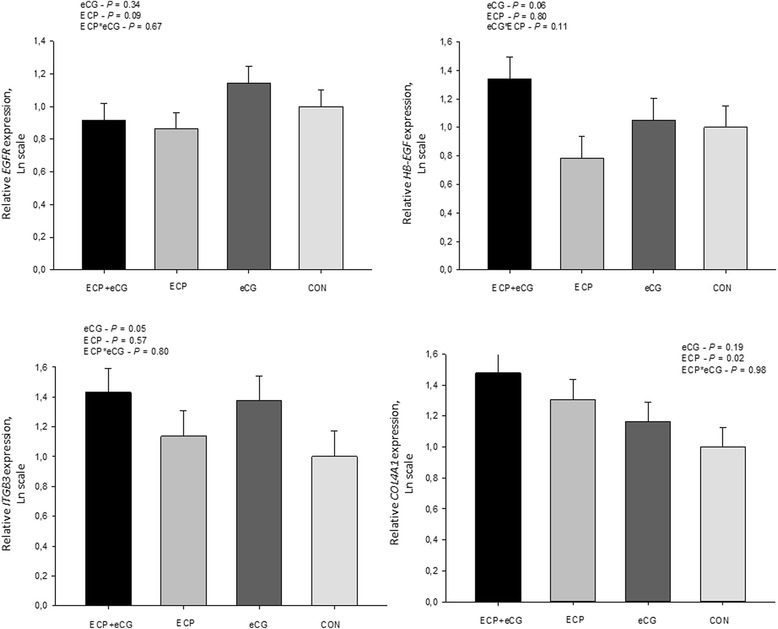
Comparison of gene expression between suckled anestrous beef cows receiving 1 mg of estradiol cipionate (ECP) and/or 400 IU of equine chorionic gonadotropin (eCG) at onset of the proestrus [CON (n = 11), ECP (n = 11), eCG (n = 12) and ECP + ECG (n = 11)]. The amounts of *EGFR*, *HBEGF*, *ITGB3,* and *COL4A1* transcripts are expressed in relation to control (CON) untreated cows. Expression values were normalized by the geometric mean of *GAPDH*, *ACTB,* and *RPS18*. The *P* values refer to comparisons made for each gene between groups (effects of ECP, eCG and interaction between ECP and eCG)

There were no interactions (*P* > 0.10) between ECP and eCG on the expression of the transcripts evaluated. ECP treatment induced greater endometrial abundance of *PTCH2* (*P* = 0.07) and *COL4A1* (*P* = 0.02) genes, whereas it reduced *EGFR* (*P* = 0.09) gene expression (Figs. 4 and 5). The ECP treatment did not affect gene expression of *ESR1* (*P* = 0.90), *ESR2* (*P* = 0.61), *HB-EGF* (*P* = 0.80) and *ITGB3* (*P* = 0.57). On the other hand, eCG treatment induced greater endometrial abundance of *HB-EGF* (*P* = 0.06), *ESR2* (*P* = 0.09), and *ITGB3* (*P* = 0.05) genes, whereas reduced the gene expression of *ESR1* (*P* = 0.05). Supplementation with eCG did not alter expression of *EGFR* (*P* = 0.34), *PTCH2* (*P* = 0.31) and *COL4A1* (*P* = 0.19). Additionally, expression of *PGR* was not altered by either ECP (*P* = 0.51) or eCG (*P* = 0.25) treatments.

## Discussion

The present study investigated the impact of hormonal manipulation of proestrus on ovarian response and on uterine transcriptome 6 d post-TAI. The most relevant observations from this study are: 1) ECP treatment improves occurrence of estrus and pregnancy per AI, whereas eCG treatment enhances final follicular growth, size of ovulatory follicle, ovulation rate and subsequent P4 concentration, 2) the endometrial transcriptional profile is regulated by ECP supplementation and cell proliferation was one of the overrepresented gene ontology terms; 3) selected candidate genes with altered expression further support an ECP effect on cellular proliferation and tissue morphology.

Synchronized cows displaying estrus before TAI exhibited larger dominant follicles, greater E2 concentration during the proestrus/estrus, greater luteal function on the subsequent estrus cycle, and greater conception rate when compared to cows that did not display estrus [6, 25, 42–44]. In agreement, exogenous ECP treatment at the onset of proestrus improved the proportion of suckled beef cows displaying estrus, determining greater pregnancy outcomes following TAI than non-ECP treated cows [25, 26], similar to what was observed in the present study. Furthermore, the eCG treatment at onset of the proestrus was effective to increase conception rates in suckled beef cows [1, 5, 29, 30]. Also corroborating with the present results, eCG-treated cows presented greater final follicular growth, follicular diameter at TAI, ovulation rate and plasma P4 concentration on subsequent diestrus [5, 26, 29, 30]. Therefore, the hormonal therapies established in the present study may be considered a pro-fertility model for suckled anestrous beef cows and potentially allow the establishment of two distinct periovulatory endocrine milieus, that are associated with an uterine environment of better receptivity. Specifically, it was expected that ECP-treated cows present greater periovulatory E2 concentration due to the exogenous estradiol administration. Additionally, those cows treated with eCG also presented greater concentrations of E2 during proestrus/estrus due to endogenous estradiol from a healthy larger DF at TAI, in addition to presenting greater concentrations of P4 during early diestrus.

Unexpectedly, transcriptome analysis of D 6 endometrium from cows treated or not with ECP did not reveal dramatic differences of gene expression patterns. Our model was unique in selecting for cows displaying estrus behavior in ECP-treated group versus not displaying estrus behavior in the control group. Estrus behavior is associated with higher pregnancy rates [6, 25, 43, 44]. Global transcriptome analysis of D 14 endometrium from high fertility heifers compared to low fertility ones did not reveal substantial differences [45]. Another study using a similar criterion for high and low fertility revealed that D 7 endometrium presented 417 DEG, however, most of the DEG exhibited fold change between 1.0 and 2.0 [46]. These results are in agreement with our data showing that endometrial gene expression is not dramatically different between groups with contrasting fertility; however, it is important to point out that half of the samples came from pregnant animals, whereas the other half came from non-pregnant cows in both ECP or control groups.

Estradiol levels are higher after ECP administration [28, 42, 47] and estrus behavior is correlated with estradiol levels [42, 48]; however, we did not quantify estradiol plasma concentrations. It was observed in ovariectomized cows that estradiol benzoate injection alters global gene expression of the endometrium when compared to a control group or progesterone treatment; whereas a combined estradiol and progesterone group shows data closer to estradiol treatment, suggesting that estradiol counteracts progesterone effects [49]. In our model, progesterone is the dominant steroid hormone at the time of sample collection; however its impact on gene expression is likely influenced by the previous exposure to estradiol.

We have observed previously that the endometrial tissue of cows ovulating larger follicles expressed markers of proliferative activity earlier than cows ovulating smaller follicles [8]. Similarly, gene expression changes suggesting reduction of proliferative activity and transition to a biosynthetic phenotype were also hastened in cows with larger ovulatory follicles. Larger follicles led to increased estradiol concentrations during proestrus and greater progesterone concentrations during early diestrus [8, 14]. Functional enrichment analysis using DAVID identified gene ontology terms associated with regulation of cell proliferation such as pathways in cancer and small cell lung cancer. Similarly, endometrial gene expression at D 7 in one estrous cycle prior to embryo transfer revealed enrichment of GO-terms cell cycle and anti-apoptosis in cows that successfully established pregnancy [50]. It is noteworthy that the above mentioned studies obtained samples from non-lactating cyclic cows [8] or heifers [50], whereas in the present study all cows were lactating and in anestrus. Additionally, assessment of the expression of proliferation-related candidate genes showed that *PTCH*2 and *COL4A1* were induced by ECP treatment, whereas *EGFR* expression was suppressed. *PTCH2* is a membrane receptor, member of the Hedgehog signaling pathway [51], and has been associated with proliferation-related disorders such as endometriosis and ovarian carcinoma [52], playing a role as a tumor suppressor gene [53]. *COL4A1* encodes a type IV collagen protein that is an integral component of basement membranes [54]. In the endometrium, the breakdown of the basement membrane as well as increased expression of *COL4A1* have been related with inhibition of angiogenesis and reduced tumor growth [55]. In addition, the oncogene *EGFR*, which is associated with growth of placental tissue [56], was downregulated by ECP-treatment suggesting a suppression of the endometrial ability to respond to mitogenic stimuli. The collective interpretation of these data is that estrogenic stimulus given by ECP induced a non-proliferative status on D6 endometrium. Such findings are consistent with our previous report, in which ovulation of a larger follicle (associated with greater proestrus and estrus plasma concentrations of estradiol) inhibited proliferation in both luminal and glandular epithelial cells on D 7 endometrium [8]. Importantly, such regulation occurred despite similar plasma concentrations of P4 between animals that received an did not receive ECP.

The most remarkable eCG-induced changes in gene expression were associated to E2 signaling. Indeed, transcript abundance was greater for *ESR2* and lesser for *ESR1* in eCG-treated cows than No eCG-treated cows, suggesting the establishment of a transition phase, from proliferative to secretory. The recognized proliferative role of estrogens in the female reproductive tract appears to be mediated by *ESR1* [57]. After estrus, *ESR1* abundance decreases and reaches nadir endometrium concentrations during the mid-luteal phase of the estrous cycle [58]. In contrast, uterine *ESR2* expression is positively associated with the increasing P4 concentration observed from early to mid diestrus. The greater abundance of *ESR2* expression found in eCG treated cows could be justified by the positive effect of eCG on P4 concentration during early diestrus. Altogether, these results suggest that the endometrium of suckled anestrous cows at Day 6 receiving either ECP or eCG is still transitioning from a proliferative to a secretory state, as previously reported [8].

## Conclusions

Supplementation with ECP or eCG at onset of the synchronized proestrus of suckled anestrous beef cows significantly influence the ovarian responses; however, the impact on global uterine gene expression is discrete, presenting few DEG that are associated with ceasing cell proliferation. Such phenotype is consistent with the beginning of the secretory phase of the endometrium, required to support conceptus growth and survival.

## Additional files

Additional file 1: Table S1. Number of reads from all samples from suckled cows receiving (ECP) or not (CON) 1 mg of ECP at the onset of the proestrous (DOCX 14 kb)

Additional file 2: Table S2. Differential gene expression results. BaseMean is the average of all samples expression profile after normalization; lfcSE – standard error from log2FoldChange; padj – *P*-value adjusted after correction of BH-FDR for multiple tests (DOCX 26 kb)

Additional file 3: Table S3. Bio-samples and Experiment accession numbers of the Raw reads resulted from the RNAseq of endometrial biopsis in the SRA data base (DOCX 20 kb)

## Abbreviations

ACTB: actin, Beta
ADAM9: ADAM metallopeptidase domain 9
ANOVA: Analysis of variance
ATP5F1: ATP Synthase, H+ Transporting, Mitochondrial Fo Complex Subunit B1
ATP5J: ATP Synthase, H+ Transporting, Mitochondrial Fo Complex Subunit F6
BCS: Body contition score
CEACAM1: Carcinoembryonic antigen-related cell adhesion molecule 1
CL: Corpus luteum
COL4A1: Collagen, type IV, alpha 1
CON: Control
Cq: Quantification cycle
DAVID: Database for Annotation, Visualization, and Integrated Discovery
DEG: Differentially expressed genes
DF: Dominant follicle
E2: Estradiol
eCG: Equine chorionic gonadotropin
ECP: Estradiol cypionate
EDTA: Ethylenediaminetetraacetic acid
EEF1D: Eukaryotic translation elongation factor 1 delta
EGFR: Epidermal growth factor receptor
ESR1: Estrogen receptor alpha
ESR2: Estrogen receptor beta
GABARAP: GABA (A) receptor-associated protein
GAPDH: Glyceraldehyde-3-Phosphate Dehydrogenase
GEMIN7: Gem nuclear organelle associated protein 7
GEO: NCBI’s gene expression omnibus
GnRH: Gonadotropin-releasing hormone
GO: Gene ontology
GPI: Glucose-6-Phosphate Isomerase
HB-EGF: Heparin-binding EGF-like growth factor
IDH3B: Isocitrate dehydrogenase 3 (NAD(+)) Beta
IM: Intramuscular
ITGB3: Integrin, beta 3
LAMC3: Laminin Subunit Gamma 3
LN: Natural logarithm
NDUFB3: NADH:Ubiquinone Oxidoreductase Subunit B3
P4: Progesterone
PEPD: Peptidase D
PGR: Progesterone receptor
PIAS1: Protein Inhibitor Of Activated STAT 1
PIK3R3: Phosphoinositide-3-Kinase regulatory subunit 3
PTCH1: Patched 1
PTCH2: Patched homolog 2
qPCR: Quantitative real time PCR
RIN: RNA integrity number
RNAseq: RNA sequencing
RPS18: Ribosomal Protein S18
RPS2: Ribosomal protein S2
SEM: Standard error of the mean
SG100G: Calcium binding protein G
SNRPD2: Small nuclear ribonucleoprotein D2 polypeptide
SRA: Sequence read archive
STRAP: Serine/Threonine Kinase Receptor Associated Protein
TAI: Timed artificial insemination
