# Supplementary material for: Impact of hormonal modulation at proestrus on ovarian responses and uterine gene expression of suckled anestrous beef cows
Source: J Anim Sci Biotechnol. 2017 Nov 1;8:79. doi: 10.1186/s40104-017-0211-3 (PMC5664832; doi:10.1186/s40104-017-0211-3)
Supplement: Supplementary file 1 — Number of reads from all samples from suckled cows receiving (ECP) or not (CON) 1 mg of ECP at the onset of the proestrous (DOCX 14 kb) [file 40104_2017_211_MOESM3_ESM.docx]

Additional file 3: **Table S3** Bio-samples and Experiment accession numbers of the Raw reads resulted from the RNAseq of endometrial biopsis in the SRA data base.

| Cow ID | BioSample | Experiment number |
| --- | --- | --- |
| 1814 | SAMN03481149 | SRX992523 |
| B1042 | SAMN03481150 | SRX992524 |
| E0040 | SAMN03481151 | SRX992525 |
| E0406 | SAMN03481152 | SRX992526 |
| E0819 | SAMN03481153 | SRX992527 |
| X228 | SAMN03481154 | SRX992528 |
| B0646 | SAMN03481155 | SRX992529 |
| C317 | SAMN03481156 | SRX992530 |
| D160 | SAMN03481157 | SRX992531 |
| E0952 | SAMN03481158 | SRX992532 |
| E1010 | SAMN03481159 | SRX992533 |
| X156 | SAMN03481160 | SRX992534 |
