# Supplementary material for: Impact of hormonal modulation at proestrus on ovarian responses and uterine gene expression of suckled anestrous beef cows
Source: J Anim Sci Biotechnol. 2017 Nov 1;8:79. doi: 10.1186/s40104-017-0211-3 (PMC5664832; doi:10.1186/s40104-017-0211-3)
Supplement: Supplementary file 2 — Differential gene expression results. BaseMean is the average of all samples expression profile after normalization; lfcSE – standard error from log2FoldChange; padj – P-value adjusted after correction of BH-FDR for multiple tests (DOCX 26 kb) [file 40104_2017_211_MOESM2_ESM.docx]

Additional file 2: **Table S2.** Differential gene expression results. BaseMean is the average of all samples expression profile after normalization; lfcSE – standard error from log2FoldChange; padj – p-value adjusted after correction of BH-FDR for multiple tests.

| **Ensemble ID** | **Gene Symbol** | **baseMean** | **log2FoldChange** | **lfcSE** | **P adj** |
| --- | --- | --- | --- | --- | --- |
| **Up-regulated ECP** |  |  |  |  |  |
| ENSBTAG00000017020 | S100G | 3843.0082 | -0.9121 | 0.1757 | 0.0016 |
| ENSBTAG00000047165 | KRT9 | 282.8445 | -1.3610 | 0.2693 | 0.0022 |
| ENSBTAG00000008950 | KIAA1456 | 40.5943 | -0.8304 | 0.1719 | 0.0041 |
| ENSBTAG00000007371 | SCAMP1 | 142.3402 | -0.6159 | 0.1313 | 0.0068 |
| ENSBTAG00000010877 | ARMC12 | 50.1830 | -1.0028 | 0.2235 | 0.0084 |
| ENSBTAG00000019587 | PI15 | 603.3697 | -0.9345 | 0.2072 | 0.0084 |
| ENSBTAG00000016078 | STXBP4 | 94.3395 | -0.6970 | 0.1528 | 0.0084 |
| ENSBTAG00000014246 | CENPH | 116.1886 | -0.5988 | 0.1320 | 0.0084 |
| ENSBTAG00000021445 | ST8SIA4 | 313.2047 | -0.5119 | 0.1139 | 0.0084 |
| ENSBTAG00000006136 | CDA | 46.2895 | -1.0408 | 0.2345 | 0.0097 |
| ENSBTAG00000023851 | FAM102A | 218.9631 | -0.5980 | 0.1362 | 0.0110 |
| ENSBTAG00000015909 | PDE8A | 466.9962 | -0.3999 | 0.0913 | 0.0110 |
| ENSBTAG00000011833 | GRIA3 | 208.1615 | -0.8653 | 0.2024 | 0.0160 |
| ENSBTAG00000032558 | TTC7A | 469.2492 | -0.7327 | 0.1733 | 0.0160 |
| ENSBTAG00000005615 | CEACAM1 | 3244.4201 | -0.6543 | 0.1532 | 0.0160 |
| ENSBTAG00000020315 | DENND5B | 143.6442 | -0.6197 | 0.1462 | 0.0160 |
| ENSBTAG00000031941 | PEPD | 5090.3997 | -0.6038 | 0.1419 | 0.0160 |
| ENSBTAG00000010244 | CLIC5 | 272.0482 | -0.7875 | 0.1868 | 0.0162 |
| ENSBTAG00000015930 | DNAL1 | 82.5170 | -0.6610 | 0.1573 | 0.0165 |
| ENSBTAG00000004172 | NA | 331.7751 | -0.3609 | 0.0879 | 0.0233 |
| ENSBTAG00000009862 | NA | 13.4794 | -1.0900 | 0.2728 | 0.0284 |
| ENSBTAG00000044126 | SNTB1 | 222.8120 | -0.7563 | 0.1890 | 0.0284 |
| ENSBTAG00000044009 | PPP1R1C | 189.3619 | -0.9883 | 0.2481 | 0.0289 |
| ENSBTAG00000004104 | RUNX2 | 85.5551 | -0.9561 | 0.2422 | 0.0323 |
| ENSBTAG00000026497 | LOC618369 | 759.5001 | -0.5313 | 0.1349 | 0.0323 |
| ENSBTAG00000024137 | PTCH2 | 1301.3648 | -0.6054 | 0.1548 | 0.0344 |
| ENSBTAG00000014564 | NA | 257.0933 | -0.3437 | 0.0885 | 0.0366 |
| ENSBTAG00000016103 | CBFB | 437.7047 | -0.3961 | 0.1029 | 0.0396 |
| ENSBTAG00000006008 | CAMSAP1 | 888.7784 | -0.2369 | 0.0615 | 0.0396 |
| ENSBTAG00000032839 | LRCH3 | 356.5190 | -0.4670 | 0.1222 | 0.0419 |
| ENSBTAG00000025659 | ZNF618 | 99.2364 | -0.5616 | 0.1474 | 0.0422 |
| ENSBTAG00000018951 | TBC1D9 | 281.0017 | -0.3394 | 0.0890 | 0.0422 |
| ENSBTAG00000021343 | ARHGEF12 | 809.1060 | -0.4111 | 0.1083 | 0.0438 |
| ENSBTAG00000005379 | SLC41A1 | 141.8958 | -0.4273 | 0.1129 | 0.0453 |
| ENSBTAG00000008734 | PM20D1 | 364.7435 | -0.9322 | 0.2470 | 0.0461 |
| ENSBTAG00000011847 | ASPN | 911.3242 | -0.6446 | 0.1735 | 0.0551 |
| ENSBTAG00000004168 | STON1 | 976.0621 | -0.4111 | 0.1106 | 0.0551 |
| ENSBTAG00000025494 | PAQR8 | 408.3517 | -0.7922 | 0.2136 | 0.0558 |
| ENSBTAG00000003701 | VASH2 | 117.6533 | -0.7892 | 0.2140 | 0.0572 |
| ENSBTAG00000015333 | TAF2 | 724.4316 | -0.2886 | 0.0790 | 0.0633 |
| ENSBTAG00000038224 | GDF7 | 137.6003 | -0.7091 | 0.1944 | 0.0636 |
| ENSBTAG00000015220 | EIF2C4 | 247.6697 | -0.4162 | 0.1148 | 0.0675 |
| ENSBTAG00000014310 | HEATR5B | 944.3612 | -0.3298 | 0.0912 | 0.0675 |
| ENSBTAG00000019725 | UBE4A | 885.0834 | -0.2799 | 0.0775 | 0.0675 |
| ENSBTAG00000000859 | SLC38A1 | 316.7010 | -0.6357 | 0.1764 | 0.0679 |
| ENSBTAG00000024657 | ZFP91 | 449.8490 | -0.2748 | 0.0768 | 0.0699 |
| ENSBTAG00000007778 | TRPM3 | 378.9482 | -0.7223 | 0.2045 | 0.0764 |
| ENSBTAG00000007807 | NA | 1324.6832 | -0.3900 | 0.1102 | 0.0764 |
| ENSBTAG00000010286 | NPAT | 983.4942 | -0.3766 | 0.1066 | 0.0764 |
| ENSBTAG00000002997 | ADAMTSL1 | 66.3420 | -0.7130 | 0.2042 | 0.0807 |
| ENSBTAG00000018186 | PDXK | 1684.1771 | -0.4882 | 0.1397 | 0.0807 |
| ENSBTAG00000020505 | SBNO1 | 405.9688 | -0.3544 | 0.1017 | 0.0809 |
| ENSBTAG00000013108 | HK2; LOC614107 | 102.2840 | -0.8450 | 0.2437 | 0.0846 |
| ENSBTAG00000000942 | SIPA1L2 | 594.4270 | -0.4460 | 0.1289 | 0.0855 |
| ENSBTAG00000017455 | ADAM9 | 647.6932 | -0.3413 | 0.0990 | 0.0872 |
| ENSBTAG00000019983 | ASXL2 | 313.8561 | -0.4458 | 0.1295 | 0.0875 |
| ENSBTAG00000009903 | PTCH1 | 632.7424 | -0.6009 | 0.1763 | 0.0919 |
| ENSBTAG00000005564 | PIAS1 | 478.6505 | -0.2490 | 0.0731 | 0.0919 |
| ENSBTAG00000022381 | TET3 | 78.6025 | -0.5727 | 0.1684 | 0.0927 |
| ENSBTAG00000009426 | EZH2 | 238.5970 | -0.5745 | 0.1696 | 0.0960 |
| ENSBTAG00000006869 | PHF20L1 | 319.0677 | -0.3096 | 0.0923 | 0.1050 |
| ENSBTAG00000017473 | Bt.54919 | 2137.2094 | -0.6364 | 0.1908 | 0.1071 |
| ENSBTAG00000011322 | HIPK1 | 706.1320 | -0.4553 | 0.1362 | 0.1071 |
| ENSBTAG00000002979 | PIK3R3 | 263.0200 | -0.2942 | 0.0879 | 0.1071 |
| ENSBTAG00000031402 | ASXL1 | 773.1369 | -0.2299 | 0.0689 | 0.1071 |
| ENSBTAG00000035018 | ZCCHC12 | 75.1708 | -0.6697 | 0.2015 | 0.1084 |
| ENSBTAG00000039766 | Bt.21928 | 383.5956 | -0.2482 | 0.0747 | 0.1084 |
| ENSBTAG00000048213 | PTCH1 | 185.2887 | -0.6647 | 0.2017 | 0.1090 |
| ENSBTAG00000003806 | ECM1 | 221.8479 | -0.6363 | 0.1922 | 0.1090 |
| ENSBTAG00000018744 | MGAT5 | 163.2504 | -0.5285 | 0.1595 | 0.1090 |
| ENSBTAG00000009267 | UHRF1BP1 | 147.9620 | -0.4851 | 0.1466 | 0.1090 |
| ENSBTAG00000003221 | NA | 477.1766 | -0.3594 | 0.1089 | 0.1090 |
| ENSBTAG00000011292 | LZTR1 | 676.1647 | -0.2536 | 0.0766 | 0.1090 |
| **Down-regulated ECP** |  |  |  |  |  |
| ENSBTAG00000032774 | C28H10orf116 | 730.9029 | 0.7802 | 0.1450 | 0.0011 |
| ENSBTAG00000009535 | RPS2 | 19615.9321 | 0.3138 | 0.0644 | 0.0041 |
| ENSBTAG00000011263 | EIF6 | 1030.4914 | 0.2519 | 0.0556 | 0.0084 |
| ENSBTAG00000012760 | NDUFB3 | 496.7634 | 0.4555 | 0.1006 | 0.0084 |
| ENSBTAG00000006441 | ATP5F1 | 1982.5894 | 0.2481 | 0.0584 | 0.0160 |
| ENSBTAG00000033395 | C25H16orf91 | 648.9370 | 0.2543 | 0.0619 | 0.0233 |
| ENSBTAG00000008394 | MYL3 | 17.0833 | 1.1227 | 0.2744 | 0.0238 |
| ENSBTAG00000006416 | EMC4 | 1055.7117 | 0.1913 | 0.0475 | 0.0273 |
| ENSBTAG00000000505 | CCBL2 | 347.4646 | 0.4728 | 0.1174 | 0.0273 |
| ENSBTAG00000038527 | C14H8orf59 | 207.6404 | 0.5089 | 0.1259 | 0.0273 |
| ENSBTAG00000032122 | NA | 45.5819 | 0.8708 | 0.2162 | 0.0273 |
| ENSBTAG00000031383 | C7H19orf77 | 20.1225 | 1.0811 | 0.2690 | 0.0273 |
| ENSBTAG00000003878 | ZUFSP | 311.7793 | 0.3136 | 0.0796 | 0.0323 |
| ENSBTAG00000020524 | UPK3BL | 245.9342 | 0.9985 | 0.2553 | 0.0344 |
| ENSBTAG00000005414 | NA | 11523.9450 | 0.3947 | 0.1014 | 0.0360 |
| ENSBTAG00000023283 | AMICA1 | 135.4681 | 0.5901 | 0.1528 | 0.0392 |
| ENSBTAG00000008541 | MGST1 | 1047.7914 | 0.7447 | 0.1947 | 0.0419 |
| ENSBTAG00000012177 | SNRPD2 | 1363.7789 | 0.2795 | 0.0751 | 0.0551 |
| ENSBTAG00000007780 | CAMK1 | 368.7778 | 0.4803 | 0.1300 | 0.0565 |
| ENSBTAG00000009842 | CRYM | 52.0086 | 0.9021 | 0.2439 | 0.0565 |
| ENSBTAG00000045538 | LOC100335806 | 195.9250 | 0.4407 | 0.1202 | 0.0611 |
| ENSBTAG00000000605 | ATP5J | 1440.8555 | 0.3211 | 0.0889 | 0.0675 |
| ENSBTAG00000002623 | LOC100850526; SAMSN1 | 101.7328 | 0.6136 | 0.1693 | 0.0675 |
| ENSBTAG00000001144 | NAA20 | 908.4684 | 0.2894 | 0.0802 | 0.0678 |
| ENSBTAG00000018809 | RANGRF | 199.9363 | 0.3599 | 0.1000 | 0.0685 |
| ENSBTAG00000047845 | NA | 658.8895 | 0.3192 | 0.0893 | 0.0699 |
| ENSBTAG00000010668 | GEMIN7 | 111.4369 | 0.3877 | 0.1087 | 0.0699 |
| ENSBTAG00000021635 | NUDT17 | 325.7542 | 0.4058 | 0.1137 | 0.0699 |
| ENSBTAG00000047136 | RPL39; LOC100297075 | 4879.3196 | 0.4270 | 0.1193 | 0.0699 |
| ENSBTAG00000034885 | RGCC | 135.8677 | 0.6831 | 0.1906 | 0.0699 |
| ENSBTAG00000010413 | TMEM169 | 64.4079 | 0.9480 | 0.2657 | 0.0699 |
| ENSBTAG00000012589 | HSPE1 | 1648.1084 | 0.2769 | 0.0779 | 0.0720 |
| ENSBTAG00000018363 | RBM48 | 206.9243 | 0.3623 | 0.1028 | 0.0766 |
| ENSBTAG00000020319 | ALOX5 | 136.1630 | 0.4818 | 0.1366 | 0.0766 |
| ENSBTAG00000046103 | NA | 9.0222 | 0.9578 | 0.2720 | 0.0766 |
| ENSBTAG00000014883 | GABARAP | 5672.5725 | 0.2119 | 0.0603 | 0.0774 |
| ENSBTAG00000015522 | MRPS31 | 256.5569 | 0.3603 | 0.1026 | 0.0774 |
| ENSBTAG00000003462 | ZNF135 | 112.5112 | 0.4495 | 0.1285 | 0.0806 |
| ENSBTAG00000005309 | TGDS | 226.2696 | 0.3142 | 0.0901 | 0.0809 |
| ENSBTAG00000002951 | CD244 | 27.4723 | 0.7143 | 0.2059 | 0.0846 |
| ENSBTAG00000014643 | EEF1D | 3740.9678 | 0.2674 | 0.0774 | 0.0861 |
| ENSBTAG00000037470 | NA | 3956.8156 | 0.4056 | 0.1175 | 0.0870 |
| ENSBTAG00000009689 | RWDD1 | 679.6372 | 0.2985 | 0.0867 | 0.0875 |
| ENSBTAG00000008370 | NA | 473.5569 | 0.2944 | 0.0859 | 0.0913 |
| ENSBTAG00000008065 | CDPF1 | 216.5052 | 0.3984 | 0.1163 | 0.0913 |
| ENSBTAG00000020496 | C23H6orf62 | 3045.6441 | 0.2325 | 0.0680 | 0.0916 |
| ENSBTAG00000007523 | STX4 | 473.8733 | 0.2828 | 0.0828 | 0.0916 |
| ENSBTAG00000006694 | CXCL14 | 383.3199 | 0.7995 | 0.2340 | 0.0916 |
| ENSBTAG00000031648 | C18H19orf33 | 321.5246 | 0.6729 | 0.1975 | 0.0919 |
| ENSBTAG00000014175 | STRAP | 1683.7075 | 0.1736 | 0.0511 | 0.0934 |
| ENSBTAG00000014695 | LSMD1 | 503.1692 | 0.2063 | 0.0613 | 0.1038 |
| ENSBTAG00000021965 | SUB1 | 2594.5113 | 0.2647 | 0.0788 | 0.1044 |
| ENSBTAG00000005028 | HIRIP3 | 289.5592 | 0.2674 | 0.0801 | 0.1071 |
| ENSBTAG00000014423 | RPL22 | 5723.1157 | 0.3640 | 0.1090 | 0.1071 |
| ENSBTAG00000018813 | IDH3B | 1617.9981 | 0.2831 | 0.0852 | 0.1084 |
| ENSBTAG00000013866 | RPS27 | 2803.5707 | 0.4255 | 0.1279 | 0.1084 |
| ENSBTAG00000021288 | PSMB4 | 2007.8008 | 0.2018 | 0.0608 | 0.1090 |
| ENSBTAG00000003607 | FAM20C | 631.1476 | 0.3027 | 0.0919 | 0.1090 |
| ENSBTAG00000021301 | ACSF2 | 1726.5727 | 0.3281 | 0.0996 | 0.1090 |
| ENSBTAG00000006396 | GPI | 2900.1266 | 0.3577 | 0.1082 | 0.1090 |
| ENSBTAG00000010426 | XRCC5 | 536.2651 | 0.6006 | 0.1821 | 0.1090 |
| ENSBTAG00000006821 | NA | 522.2814 | 0.6814 | 0.2063 | 0.1090 |
