# Supplementary material for: Impact of hormonal modulation at proestrus on ovarian responses and uterine gene expression of suckled anestrous beef cows
Source: J Anim Sci Biotechnol. 2017 Nov 1;8:79. doi: 10.1186/s40104-017-0211-3 (PMC5664832; doi:10.1186/s40104-017-0211-3)
Supplement: Supplementary file 3 — Bio-samples and Experiment accession numbers of the Raw reads resulted from the RNAseq of endometrial biopsis in the SRA data base (DOCX 20 kb) [file 40104_2017_211_MOESM1_ESM.docx]

Additional file 1: **Table S1.** Number of reads from all samples from suckled cows receiving (ECP) or not (CON) 1mg of ECP at the onset of the proestrous.

| Cow Id. | Group | Number of raw reads^1^ | Number of reads after filtering^2^ | Number of mapped reads^3^ | Number of mapped paired reads^4^ | Number of singletons^5^ | % mapped reads^6^ |
| --- | --- | --- | --- | --- | --- | --- | --- |
| E1010 | ECP | 22420008 | 17506266 | 15134829 | 8923408 | 2621269 | 86.5 |
| X156 | ECP | 25895472 | 22420554 | 20892565 | 16829460 | 1315305 | 93.2 |
| D160 | ECP | 30358500 | 26194522 | 24087314 | 19956258 | 1565380 | 92.0 |
| B0646 | ECP | 30678230 | 24662446 | 20830906 | 20830906 | 3238296 | 84.5 |
| C317 | ECP | 33077598 | 26839302 | 22999465 | 16787414 | 3508161 | 85.7 |
| E0952 | ECP | 24005780 | 20834790 | 19151177 | 15705178 | 1262607 | 91.9 |
| E0040 | CON | 31306148 | 25255034 | 20828184 | 14730270 | 3822828 | 82.5 |
| E0406 | CON | 31900030 | 25753722 | 21552085 | 15647644 | 3699979 | 83.7 |
| E0819 | CON | 27075074 | 23518146 | 22075007 | 17473114 | 1361155 | 93.9 |
| 1814 | CON | 24503324 | 21302684 | 19913174 | 16162692 | 1211000 | 93.5 |
| B1042 | CON | 31408510 | 25403572 | 21772305 | 16000958 | 3341991 | 85.7 |
| X228 | CON | 22330398 | 19183928 | 17739755 | 14347750 | 1129057 | 92.5 |

^1^Number of raw reads – total number of reads obtained from the sequencing;

^2^Number of reads after filtering – number of reads after filtering out contaminants and low quality sequences;

^3^Number of mapped reads – total number of reads that mapped against the bovine genome;

^4^Number of mapped paired reads – reads PE that mapped on the same region or chromosome;

^5^Number of singletons – number of reads which mapped without its pair;

^6^ % mapped reads – proportion of reads mapped considering the filtered reads.
